# Outcome and prognostic factors after lung transplantation for bronchiectasis other than cystic fibrosis

**DOI:** 10.1186/s12890-021-01634-z

**Published:** 2021-08-13

**Authors:** Takashi Hirama, Fumiko Tomiyama, Hirotsugu Notsuda, Tatsuaki Watanabe, Yui Watanabe, Hisashi Oishi, Yoshinori Okada

**Affiliations:** 1 Department of Thoracic Surgery, Institute of Development, Aging and Cancer, Tohoku University, 4-1 Seiryo-machi, Sendai, Miyagi Japan; 2 Division of Organ Transplantation, Tohoku University Hospital, 1-1 Seiryo-machi, Sendai, Miyagi Japan

**Keywords:** Lung transplant, Bronchiectasis, *Pseudomonas aeruginosa*, Sinusitis, Chronic lung allograft dysfunction, Non-tuberculous mycobacteria

## Abstract

**Background:**

While lung transplant (LTX) can be an effective therapy to provide the survival benefit in selected populations, post-transplant outcome in LTX recipients with bronchiectasis other than cystic fibrosis (CF) has been less studied. *Pseudomonas aeruginosa*, often associated with exacerbations in bronchiectasis, is the most common micro-organism isolated from LTX recipients. We aimed to see the outcomes of patients with bronchiectasis other than CF after LTX and seek the risk factors associated with pre- and post-transplant *Pseudomonas* status.

**Methods:**

Patients who underwent LTX at Tohoku University Hospital between January 2000 and December 2020 were consecutively included into the retrospective cohort study. Pre- and post-transplant prevalence of *Pseudomonas* colonization between bronchiectasis and other diseases was reviewed. Post-transplant outcomes (mortality and the development of chronic lung allograft dysfunction (CLAD)) were assessed using a Cox proportional hazards and time-to-event outcomes were estimated using the Kaplan–Meier method.

**Results:**

LTX recipients with bronchiectasis experienced a high rate of pre- and post-transplant *Pseudomonas* colonization compared to other diseases with statistical significance (*p* < 0.001 and *p* < 0.001, respectively). Nevertheless, long-term survival in bronchiectasis was as great as non-bronchiectasis (Log-rank *p* = 0.522), and the bronchiectasis was not a trigger for death (HR 1.62, 95% CI 0.63–4.19). On the other hand, the chance of CLAD onset in bronchiectasis was comparable to non-bronchiectasis (Log-rank *p* = 0.221), and bronchiectasis was not a predictor of the development of CLAD (HR 1.88, 95% CI 0.65–5.40).

**Conclusions:**

Despite high prevalence of pre- and post-transplant *Pseudomonas* colonization, the outcome in LTX recipients with bronchiectasis other than CF was comparable to those without bronchiectasis.

**Supplementary Information:**

The online version contains supplementary material available at 10.1186/s12890-021-01634-z.

## Introduction

Bronchiectasis is a heterogeneous airway disease characterized by irreversible dilatation of bronchial lumen leading to chronic respiratory symptoms and recurrent pulmonary infections with a reduction of lung function [1, 2]. Because of poor outcome in the severe form of bronchiectasis, lung transplant (LTX) is an effective therapy to prolong the survival in the selected population [3, 4]. Despite its heterogenous etiology, cystic fibrosis (CF) is a clinically relevant cause of bronchiectasis and a common indication for LTX worldwide and its outcome following LTX has been successively reported. However, the post-transplant outcomes from bronchiectasis other than CF has been less studied. This may be because bronchiectasis other than CF has been considered as having less favorable outcomes and more complicated postoperative courses due to older age compared to CF [5, 6]. Additionally, some studies combined two bronchiectasis subgroups in a single cohort [7, 8], resulting in fewer reports regarding bronchiectasis other than CF.

CF is a common inherited disorder among Caucasians with an estimated incidence of 1 in 4500 live births in Western Europe and 1 in 4000 in North America [9], while in Japanese populations it is extremely rare, reported 1 in 350,000 [10]. Thus, further understanding post-transplant outcomes in bronchiectasis other than CF is needed to provide LTX for patients with advanced bronchiectasis in Japan.

Progression of bronchiectasis can be caused by a variety of pathogenic micro-organisms [11]. While the clinical significance of non-tuberculous mycobacteria (NTM) and *Aspergillus* is becoming recognized and their prevalence is increasing worldwide in bronchiectasis [12, 13], *Pseudomonas aeruginosa* is the most commonly isolated pathogen and related to a severe form as well as frequent exacerbations in bronchiectasis [1, 3, 14]. Similarly, *Pseudomonas* is the most frequently isolated pathogen from lung grafts as well as the sinus in LTX recipients with CF [15–17], which was currently considered as a risk factor for the worse post-transplant outcomes [18–20].

We thus hypothesized that the patients with bronchiectasis other than CF who underwent LTX likely harbor the more common *Pseudomonas* prior to transplantation and consequently the recipients could retain a high prevalence of *Pseudomonas* colonizing their airways after surgery, resulting in a higher incidence of chronic lung allograft dysfunction (CLAD) and mortality than other disorders. We therefore aimed to see the outcomes of patients with bronchiectasis other than CF after LTX and to seek the risk factors associated with pre- and post-operative *Pseudomonas* status.

## Materials and methods

### Patient population and study objectives

**Fig. 1:**
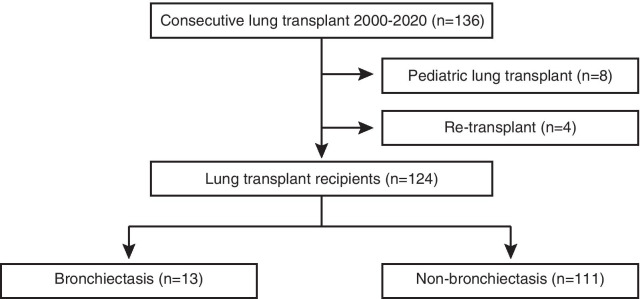
Study flow

Patients who underwent LTX at Tohoku University Hospital (TUH) between January 1st, 2000 and December 31st, 2020 were consecutively included in the retrospective cohort, with follow-up extending to December 31st, 2020 (Fig. 1). LTX recipients who were younger than 18 years old or re-transplanted were excluded from the study. Baseline data were obtained at the time of LTX, and follow-up data were collected at month 1, 2, 3 and 6, and annually post-transplant, or when clinically indicated. Surveillance bronchoscopy was not routinely scheduled in our program but performed when clinically needed. Immunosuppression, histocompatibility testing and overall management after transplantation have been previously described [21–23]. All LTX recipients received valganciclovir 900 mg daily for CMV prophylaxis for 1 year after transplantation and have been on a life-long prophylaxis with trimethoprim 80 mg-sulfamethoxazole 400 mg and itraconazole 200 mg (transplanted between 2008 and 2018) or voriconazole adjusted to target a trough concentration between 1 and 2 µg/ml (transplanted after 2018).

### Study objectives

The primary objective of the study was to see the mortality and the incidence of CLAD among LTX recipients with or without bronchiectasis and assess their risk factors. The secondary objective was to review the pre- and post-transplant prevalence of *Pseudomonas* colonization between bronchiectasis and other diseases, and seek the risk factors associated with its colonization after LTX. The tertiary objective was to observe the incidence of other pathogens including NTM and *Aspergillus* in those populations.

### Definition of variables

Radiographic morphology of bronchiectasis was assessed by two experienced respirologists and categorized according to a previous report [24]. Chronic sinusitis was defined by at least two cardinal symptoms from the following: facial discomfort, hyposmia, nasal drainage, and nasal obstruction over 12 weeks and radiographic evidence of opacification in the paranasal sinuses through computed tomography (CT) [25]. CLAD was defined as ≥ 20% of irreversible drop in FEV1 from the baseline which was confirmed two times 3 months after LTX [26].

### Microbiological assessment

Sputum, induced sputum, or bronchial washing fluid (sputum hereafter) was collected from LTX recipients at the monthly follow-up clinic or the annual hospital visit, or when respiratory symptoms were newly developed or pulmonary function was deteriorated. Sputum was sent to the microbiology laboratory at TUH, assessed for the morphologic characterization by Gram staining and acid-fast bacillus (AFB)- fluorescence microscopy, and cultured into 7 different media including sheep-blood, chocolate and Drigalski lactose for bacteria, Sabouraud and CHROMagar Candida™ (Kanto Kagaku CO. Inc., Tokyo, Japan) for fungus and AFB liquid broth and solid culture for mycobacteria. Bacteria were incubated for 48 h, filamentous fungus for 14 days and mycobacteria for 6 weeks. Microorganism was identified by the matrix-assisted laser desorption ionization-time of flight (MALDI-TOF) mass spectrometry. The threshold value for the positive culture of *Pseudomonas aeruginosa* was set for ≥ 10^3^ colony forming units (CFU)/mL [27]. *Pseudomonas* was considered colonization when cultured twice at least 3 months apart over a 12-month period [14]. Non-aeruginosa *Pseudomonas* was excluded from *Pseudomonas* colonization. Two positive cultures of NTM from sputum was regarded colonization [28], while *Aspergillus* colonization was defined by one positive culture of *Aspergillus* species from sputum [29]. Cases of apparent or sub-clinical infection due to *Pseudomonas*, NTM or *Aspergillus* were included in the colonization.

### Statistical analysis

The variables between bronchiectasis and non-bronchiectasis at the time of LTX were shown in percentage or medians (interquartile range (IQR)) as appropriate, and the difference in baseline data were assessed with chi-square or Fisher’s exact tests for categoric variables and Mann–Whitney U test for continuous variables. The cross-sectional analysis for the post-transplant outcomes were carried out based on the date on December 31st, 2020. Risk factors associated with post-transplant events were assessed using a Cox proportional hazards model. Variables considered a priori to be clinically important (age, sex, LTX procedure, LTX indication and ischemic time) and known bronchiectasis risk factors (history of pre-transplant *Pseudomonas* colonization and chronic sinusitis) were selected for analysis. Only univariate analysis was shown in result due to the small sample size of patients with bronchiectasis, while multivariate analysis was shown in supplemental data. The Kaplan–Meier method was used to model time-to-event outcomes, and differences across groups were calculated with the log-rank test. Unadjusted survival analyses were performed to avoid overfitting due to the small sample size. *p* Values of < 0.05 were considered statistically significant. Statistical analyses and graph generation were performed with GraphPad Prism 6.0 (GraphPad Software, Inc., La Jolla, CA) and StatPlus:macLE (AnalystSoft; Walnut, California, US).

## Results

### Study population and characteristics of patients with bronchiectasis

One hundred and twenty-four patients who received a LTX between January 2000 and December 2020 were serially included for analysis (Fig. 1). Median age was 45 (IQR 34-51) and 38.7% were male (Table 1). Single lung transplant was the most common surgical procedure at 67/124 (54.0%), and obstructive lung disease was the major LTX indication in 51/124 (39.5%). Chronic sinusitis was found in 19/124 (15.3%) of the recipients, connective tissue disease in 14/124 (11.3%) and history of pre-transplant *Pseudomonas* colonization in 13/124 (10.5%). Patients were divided into bronchiectasis (n = 13) and non-bronchiectasis (n = 111) groups. There was no difference in age and gender between groups, yet a bilateral lung transplant procedure was the more common LTX procedure in bronchiectasis as compared to non-bronchiectasis (*p* < 0.0001). Chronic sinusitis and *Pseudomonas* colonization were more readily found in patients with bronchiectasis compared to those without bronchiectasis (*p* < 0.0001 and *p* < 0.0001, respectively). No other difference was found in pre-transplant comorbidities between the patients with and without bronchiectasis. According to LTX procedure, ischemic time in bronchiectasis was longer than non-bronchiectasis (*p* = 0.002). Suppurative lung disease, herein synonymously bronchiectasis, accounted for 10.5% (13/124) of LTX indication in our center, and its etiology is shown in Table 2. Diffuse pan-bronchiolitis (DPB) was the major underlying disease at 5/13 (38.5%), followed by unknown etiology at 4/13 (30.8%). Two patients had bronchiectasis due to systemic inflammatory diseases and both were diagnosed with rheumatoid arthritis. No CF patients underwent LTX in our center. Bronchiectasis progressed up until the recipients received LTX, at which point the thoracic CT demonstrated cystic changes in most patients (11/13, 84.6%).

**Table 1:** Recipients' characteristics

	All patients n = 124	Bronchiectasis n = 13	Non-bronchiectasis n = 111	*p value*
Age at LTX, median (IQR)	45 (34–51)	50 (44.5–53.5)	43 (33–50)	0.062
Male, n (%)	48 (38.7%)	7 (53.8%)	41 (36.9%)	0.247
LTX procedure, n (%)				< 0.0001
Cadaveric single	67 (54.0%)	0 (0.0%)	67 (60.4%)	
Cadaveric bilateral	48 (38.7%)	12 (92.3%)	36 (32.4%)	
Living-donor	9 (7.3%)	1 (7.7%)	8 (7.2%)	
LTX indication category, n (%)				N/A
Suppurative lung disease	13 (10.5%)	13 (100%)		
Restrictive lung disease	30 (24.2%)		30 (27.0%)	
Pulmonary vascular disease	27 (21.8%)		27 (24.3%)	
Obstructive lung disease	51 (39.5%)		51 (49.9%)	
Others	3 (2.4%)		3 (2.7%)	
Chronic sinusitis, n (%)	19 (15.3%)	10 (76.9%)	9 (8.1%)	< 0.0001
Connective tissue disease, n (%)	14 (11.3%)	2 (15.4%)	12 (10.8%)	0.641
Gastroesophageal reflux disease, n (%)	10 (8.1%)	2 (15.4%)	8 (7.2%)	0.282
Diabetes	8 (6.5%)	0 (0.0%)	8 (7.2%)	0.999
Underweight (BMI < 18.5 kg/m2), n (%)	67 (54.0%)	6 (46.2%)	61 (55.0%)	0.571
History of *Pseudomonas* colonization, n (%)	13 (10.5%)	12 (92.3%)	1 (0.9%)	< 0.0001
History of NTM isolation, n (%)	5 (4.0%)	1 (7.7%)	4 (3.6%)	0.431
Ischemic time (min), median (IQR)	502 (431–666)	685 (635–734)	493 (429–643)	0.002
CMV mismatch, n (%)	21 (16.9%)	1 (8.3%)	20 (18%)	0.689

LTX, lung transplant; IQR, interquartile range; BMI, body-mass index; NTM, non-tuberculous mycobacterium; CMV, cytomegalovirus; N/A, not applicable

**Table 2:** Profile of bronchiectasis (n = 13)

A. Cause of bronchiectasis (n = 13), n (%)	
Consequence of childhood infection	1 (7.7%)
Aspiration/gastro-esophageal reflux	1 (7.7%)
Common variable immunodeficiency	0 (0.0%)
Systemic inflammatory diseases	2 (15.4%)
Cystic Fibrosis	0 (0.0%)
Primary ciliary dyskinesia	0 (0.0%)
Diffuse panbronchiolitis	5 (38.5%)
Allergic bronchopulmonary aspergillosis	0 (0.0%)
Unknown etiology	4 (30.8%)
B. Macroscopic morphology (n = 13), n (%)	
Cylindrical bronchiectasis	1 (7.7%)
Varicose bronchiectasis	1 (7.7%)
Cystic bronchiectasis	11 (84.6%)

### Outcomes of LTX recipients with bronchiectasis

**Fig. 2:**
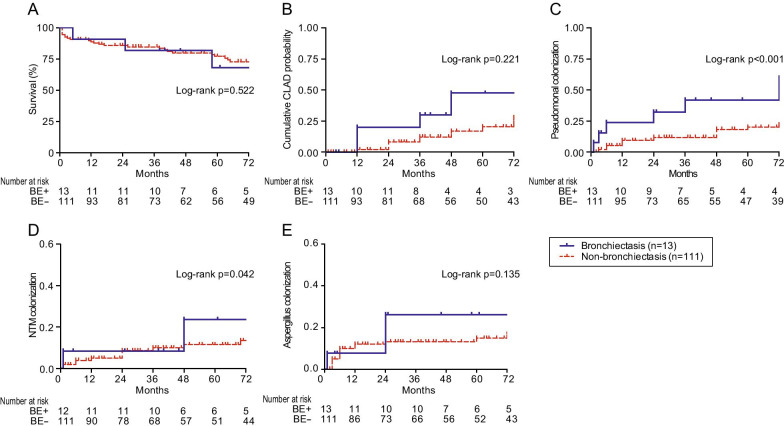
Kaplan–Meier analysis to model time-to-event outcomes in lung transplant recipients with/without bronchiectasis. **A** Percent survival, **B** cumulative incidence of CLAD probability, **C** cumulative prevalence of *Pseudomonas* colonization, **D** cumulative prevalence of NTM colonization and **E** cumulative prevalence of *Aspergillus* colonization. The number of patients at risk was depicted below the x-axis (post-transplant months). BE; bronchiectasis, CLAD; chronic lung allograft dysfunction, NTM; non-tuberculous mycobacteria

**Fig. 3:**
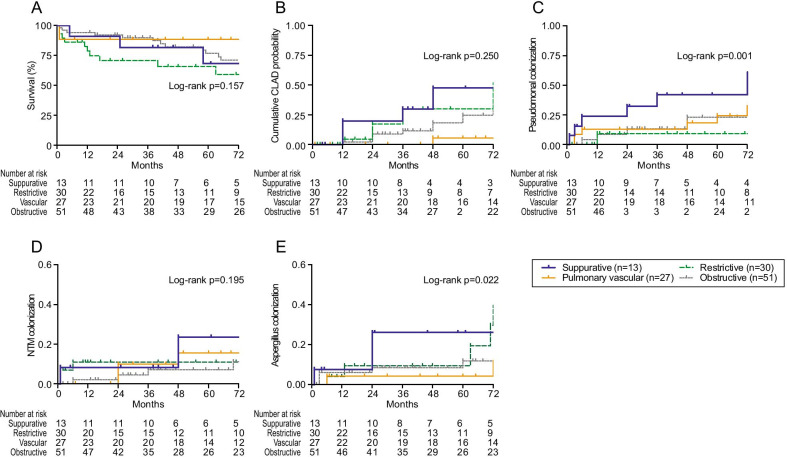
Kaplan–Meier analysis to model time-to-event outcomes in lung transplant recipients among 4 transplant categories. **A** Percent survival, **B** cumulative incidence of CLAD probability, **C** cumulative prevalence of *Pseudomonas* colonization, **D** cumulative prevalence of NTM colonization and **E** cumulative prevalence of *Aspergillus* colonization. The number of patients at risk was depicted below the x-axis (post-transplant months). BE; bronchiectasis, CLAD; chronic lung allograft dysfunction, NTM; non-tuberculous mycobacteria

Time to event outcomes between bronchiectasis (n = 13) and non-bronchiectasis (n = 111) is shown in Fig. 2. There was no survival difference in the overall study cohort between groups (Log-rank *p* = 0.522). Although the probability of CLAD development in bronchiectasis was not statistically higher than non-bronchiectasis (Log-rank *p* = 0.221), there seemed to be numerical differences between groups. On the other hand, the incidence of *Pseudomonas* colonization was significantly greater in bronchiectasis group than non-bronchiectasis (Log-rank *p* < 0.001). The chances of NTM colonization were more likely in bronchiectasis (Log-rank *p* = 0.042) rather than non-bronchiectasis, while that of *Aspergillus* was not different between the groups (Log-rank *p* = 0.135). The same analysis using Kaplan–Meier method was performed for every LTX category, including restrictive lung disease (n = 30), vascular (n = 27), obstructive (n = 51) versus suppurative (n = 13), shown in Fig. 3. The survival rate in bronchiectasis was not significantly different among the four categories (Log-rank *p* = 0.157) despite the restrictive lung disease group seemingly having a lower rate. There was no difference in time to CLAD onset among transplant categories (Log-rank *p* = 0.250), yet the cumulative CLAD probability in the vascular group was apparently lower than the suppurative group. In contrast, the incidence of *Pseudomonas* colonization after LTX was more likely in the suppurative group than other categories (Log-rank *p* = 0.01). The chances of post-transplant NTM colonization were not remarkable among LTX categories (Log-rank *p* = 0.195), whereas *Aspergillus* colonization was more seen in the suppurative group than the others in a portion of the study periods (Log-rank *p* = 0.022). The post-transplant outcomes in bronchiectasis and non-bronchiectasis were cross-sectionally analyzed (Table 3). The fraction of death (38.5% vs 27.0%, *p* = 0.515) and time to death (25 months (IQR 3–85) vs 14 months (IQR 1–58), *p* = 0.775) were not significant between groups. The leading cause of death on LTX recipients at THU was infection, responsible for 60% (3/5) in bronchiectasis and 23.3% (7/30) in non-bronchiectasis without difference in groups (*p* = 0.643).

**Table 3:** The cross-sectional analysis for outcomes in lung transplant recipients with and without bronchiectasis

	All patients n = 124	Bronchiectasis n = 13	Non-bronchiectasis n = 111	*p value*
Median time of follow-up, months (IQR)	59 (21–99)	46 (15–89)	60 (21–100)	0.504
Death, n (%)	35 (28.2%)	5 (38.5%)	30 (27.0%)	0.515
Cause of death				
Infection	10 (26.6%)	3 (60.0%)	7 (23.3%)	0.642
CLAD	9 (25.7%)	2 (40.0%)	7 (23.3%)	
Primary graft dysfunction	6 (17.1%)	0 (0.0%)	6 (20.0%)	
Cardiovascular complications	3 (8.6%)	0 (0.0%)	3 (10.0%)	
Gastrointestinal complications	2 (5.7%)	0 (0.0%)	2 (6.7%)	
Malignancy	2 (5.7%)	0 (0.0%)	2 (6.7%)	
Technical complications	1 (2.9%)	0 (0.0%)	1 (3.3%)	
Other	2 (5.7%)	0 (0.0%)	2 (6.7%)	
Median time to death, months (IQR)	15 (1–58)	25 (3–85)	14 (1–58)	0.775
CLAD, n (%)	35 (28.2%)	4 (30.8%)	31 (27.9%)	0.999
Median time to CLAD development, months (IQR)	60 (24–60)	24 (12–45)	72 (36–96)	0.036
Pseudomonas colonization, n (%)	29 (23.4%)	8 (61.5%)	21 (18.9%)	0.002
Median time to first isolation of Pseudomonas, months (IQR)	24 (6–78)	30 (4–93)	24 (9–66)	0.857

LTX, lung transplant; IQR, interquartile range

### Risk factors associated with outcomes

Risk factors for each outcome in univariable analysis using a Cox hazard model are shown in Table 4. As the two lobes were implanted into bilateral chest cavities, the living-donor transplant was categorized as bilateral transplant in the analysis. Bronchiectasis (n = 13), compared to non-bronchiectasis (n = 111), was not associated with mortality in the overall study cohort (HR 1.62, 95% CI 0.63–4.19), yet age was a risk factor to death (HR 1.03, 95% CI 1.01–1.08). Risk factors for the development of CLAD were also analyzed, showing the recipient age at the LTX (HR 1.04, 95% CI 1.01–1.08) and chronic sinusitis (HR 2.56, 95% CI 1.10–5.99) becoming predictors of CLAD onset but bronchiectasis was not associated with its development (HR 1.88, 95% CI 0.65–5.40). Similar to the finding shown in the Kaplan–Meier method, bronchiectasis was associated with post-transplant *Pseudomonas* colonization (HR 4.30, 95% CI 1.88–9.85). Additionally, the LTX procedure (bilateral vs single, HR 2.21, 95% CI 1.01–4.76), history of pre-transplant *Pseudomonas* colonization (HR 3.77, 96% CI 1.65–8.62) and chronic sinusitis (HR 2.75, 95% CI 1.21–6.28) demonstrated statistical significance for increased risk for post-transplant *Pseudomonas* colonization. There was a trend of NTM colonization in LTX recipients with bronchiectasis (HR 3.01, 95% CI 0.97–9.32) and chronic sinusitis (HR 2.69, 95% CI 0.94–97.69) in the univariable analysis but not a statistically higher chance. Although bronchiectasis was not a predictor of post-transplant *Aspergillus* colonization (HR 2.24, 95% CI 0.75–6.70), history of pre-transplant *Pseudomonas* colonization could be related to its isolation (HR 2.91, 95% CI 1.05–8.01).

**Table 4:** Hazard ratio for risk factors for mortality, development of CLAD and *Pseudomonas* colonization from univariate Cox model

Covariate	A. Risk factors for death			B. Risk factors for CLAD			C. Risk factors for *Pseudomonas* colonization		
	*p* value	HR	95% CI	*p* value	HR	95% CI	*p* value	HR	95% CI
Recipient age at LTX	0.013	1.04	1.01–1.08	0.022	1.04	1.01–1.08	0.544	1.01	0.98–1.05
Recipient sex (male vs female)	0.997	1.00	0.49–2.02	0.191	1.58	0.80–3.14	0.500	1.31	0.60–2.90
LTX procedure (bilateral vs single)	0.857	0.94	0.48–1.83	0.182	0.62	0.31–1.25	0.044	2.21	1.02–4.76
LTX indication (bronchiectasis vs others)	0.320	1.62	0.63–4.19	0.241	1.88	0.65–5.40	0.001	4.30	1.88–9.85
History of pre-transplant *Pseudomonas*	0.412	1.49	0.58–3.85	0.143	2.05	0.78–5.36	0.002	3.77	1.65–8.62
Chronic sinusitis	0.462	1.39	0.58–3.37	0.030	2.56	1.10–5.99	0.016	2.75	1.21–6.28
Ischemic time	0.999	1.00	0.99–1.01	0.320	0.999	0.99–1.01	0.933	1.00	0.99–1.01

Covariate	D. Risk factors for NTM colonization			E. Risk factors for *Aspergillus* colonization		
	*p* value	HR	95% CI	*p* value	HR	95% CI
Recipient age at LTX	0.241	1.03	0.98–1.08	0.432	1.02	0.98–1.06
Recipient sex (male vs female)	0.107	2.26	0.84–6.09	0.988	0.99	0.39–2.51
LTX procedure (bilateral vs single)	0.538	1.35	0.52–3.51	0.985	0.99	0.41–2.39
LTX indication (bronchiectasis vs others)	0.055	3.01	0.97–9.32	0.151	2.24	0.75–6.70
History of pre-transplant *Pseudomonas*	0.334	1.86	0.53–6.52	0.039	2.91	1.05–8.01
Chronic sinusitis	0.064	2.69	0.94–7.69	0.064	2.48	0.95–6.45
Ischemic time	0.576	1.00	0.99–1.01	0.800	1.00	0.99–1.01

LTX, lung transplant; CLAD, chronic lung allograft dysfunction; CI, confidence interval; HR, hazard ratio; NTM, non-tuberculous mycobacterium

## Discussion

As previously reported that *Pseudomonas* is frequently isolated in patients with bronchiectasis and LTX recipients [1, 3, 18, 30, 31], our study demonstrated that LTX recipients with bronchiectasis other than CF experienced high rate of pre- and post-transplant *Pseudomonas* colonization with statistical significance. Nevertheless, long-term survival in the bronchiectasis group was as great as the non-bronchiectasis group or other disease categories, and bronchiectasis was not an independent risk for CLAD development. Our results were consistent with other analyses that survival rate was similar between bronchiectasis (n = 42) versus other diseases requiring bilateral LTX in UK [32] and between bronchiectasis with CF (n = 42) and non-CF (= 33) in Israel [6] although isolation of *Pseudomonas aeruginosa* was common in those populations. In view of these considerations, it is conceivable that bronchiectasis, despite high prevalence of pre- and post-transplant *Pseudomonas* colonization, is not a prominent risk factor for the post-transplant mortality and the development of CLAD. However, a contradictory outcome was reported from a LTX center in Australia [33], where lower 5-year survival and more hospital admission were shown in bronchiectasis other than CF. As numerous confounding factors affect the outcomes after transplantation in this population, multivariate analysis would be helpful for further understanding of the risks in those population. To this end, the study including a large number of patients should be planned to see which variables among the individuals with bronchiectasis would have an impact on the post-transplant outcomes.

Given their ubiquitous presence in many environments, both NTM and *Aspergillus* are also frequently identified from LTX recipients but considered more unfavorably due to their pathogenic roles, and currently regarded as probable risk factors for the poor outcomes among LTX recipients [34–37]. A higher cumulative incidence of post-transplant NTM colonization was found in bronchiectasis compared to other diseases (Log-rank 0.042), whereas the post-transplant prevalence of *Aspergillus* was high in the suppurative disease rather than the other categories (Log-rank *p* = 0.022). In view of the graft and native lungs that accompany anatomic abnormalities and are constantly exposed to ubiquitous environmental micro-organisms, superinfection or double- or triple-isolation of *Pseudomonas*, NTM and *Aspergillus* is an expected consequence after LTX. However, pathogenic roles of those organisms are not clearly defined because of complicated pathogen-host interactions especially under immune-suppressants and the heterogeneous pathogenesis of bronchiectasis. Furthermore, microbiological assessment of the pathogenic aspect of those organisms is challenging as there are no validated biomarkers to distinguish infection from colonization and also the majority of LTX recipients is routinely or repeatedly on anti-microbial agents for prophylaxis or treatment. With our analysis, the post-transplant prevalence of *Pseudomonas*, NTM and *Aspergillus* was high in LTX recipients with bronchiectasis. Nevertheless, an extended study to see how those micro-organisms influence the graft function and how anti-microbial agents, together with immunosuppression, play roles in such population are needed.

A prominent feature of bronchiectasis other than CF is an involvement of chronic sinusitis with little known etiology [38]. Sinusitis is considered a reservoir for allograft colonization of micro-organisms after LTX [16, 17]. In our assessment, chronic sinusitis was an independent risk factor for CLAD (HR 2.56, 95% CI 1.10–5.99) and post-transplant *Pseudomonas* colonization (HR 2.75, 95% CI 1.21–6.28). In previous studies, sinus surgery led to an improvement in pulmonary function in LTX recipients with sinusitis [39] and reduced *Pseudomonas* colonization in CF-LTX recipients [15]. Importantly, there was a high correlation between pre-transplant sinus and post-transplant BAL cultures for *Pseudomonas* [17] and the same isolates were found in nasal lavage and BAL performed on the same visit in CF patients [16]. With those features in mind, it should be reasonable to consider the early intervention of sinus surgery prior to LTX or in the early phase after LTX, which may be capable of preventing from the development of CLAD in the specific population with chronic sinusitis. Yet, different analyses demonstrated little impact of pre-transplant sinus surgery on post-transplant recolonization of *Pseudomonas* in a CF population [40]. Thus, our next challenge is to consider the clinical trial to prospectively intervene whether sinus surgery affect the transplant outcome in patients with sinusitis.

Nonetheless, our study must be interpreted with caution and a number of limitations should be considered. First, we have insufficient sample size for further analysis. In order to seek the risk factors for outcomes, there were variables that needed to be included for the analysis, such as bronchiectasis, pre-transplant *Pseudomonas* colonization and chronic sinusitis, with which multivariate analysis should be performed. Due to shortage of the bronchiectasis patients (n = 13), the multivariate cox hazard model showed a wide confidence interval (Additional file 1: Supplemental data) and was not worth documenting. Despite the univariate analysis that lacks adjustments for comparisons or power for multivariate analysis, comparable survival rates and a high rate of pre- and post-transplant *Pseudomonas* colonization in bronchiectasis were evident from our study. A multicenter study including a large number of patients with bronchiectasis for analysis would be beneficial in seeing the outcome calculated on the basis of a multivariate analysis. Second, we were unable to analyze whether the post-transplant *Pseudomonas* led to the CLAD onset, or vice versa. An etiology between post-transplant *Pseudomonas* colonization and CLAD development is a chicken-or-egg problem and remains unexplored, yet colonized *Pseudomonas* was partially or somewhat considerably associated with developing or worsening CLAD [18, 19, 30, 31]. To understand whether the duration of one variable is a risk factor for another variable is complicated when it may occur at some time after LTX. Apart from causality that has never been proven through observational studies, the recent study from clinical practice demonstrated *Pseudomonas* eradication after LTx improved CLAD-free and graft survival and maintained pulmonary function [31]. This kind of intervention is a means to prove its complicated relationship and a feasible approach to seek how best we could provide better outcome among individuals with bronchiectasis after LTX. In addition to those above analyses, it would be intriguing to see whether pre- and post-transplant *Pseudomonas* are the same strains by genotyping [41] and how multi-drug resistant strains affect the outcome [42]. Finally, additional analysis in the details of post-transplant complications will be needed for a better understanding those populations. CLAD was determined in 4 LTX recipients with bronchiectasis other than CF in the study period, of which 2 cases were obstructive and the others restrictive in CLAD phenotype. It cannot be conclusive from such a small number of CLAD cases whether they tended to show which phenotype of allograft loss. On the other hand, a recent study demonstrated bronchiectasis rather than CF had higher rate of CLAD with infectious features than other diseases [33]. Given higher prevalence of chronic sinusitis and chances of *Pseudomonas* colonization, infectious exacerbations could be more commonly seen in LTX recipients with bronchiectasis other than CF. As the lack of those data in our study, the next challenge is to investigate the post-transplant complications in those individuals in a large-scale analysis.

## Conclusions

In conclusion, the long-term outcome in LTX recipients with the underlying disease of bronchiectasis other than CF, a representative of the suppurative lung disease in Japan, was comparable to those without bronchiectasis. Our investigation also demonstrated a similar ratio of CLAD development despite a higher chance of *Pseudomonas* colonization in bronchiectasis compared to other diseases or categories. Although multivariate analysis will be needed for the further understanding of risk factors for post-transplant outcomes, this study will be fundamental to future trials for individuals with bronchiectasis requiring LTX.

## Supplementary Information

**Additional file 1**. Risk factors for mortality, development of CLAD and Pseudomonas colonization from multivariate Cox model.

## Data Availability

The datasets used and/or analysed during the current study are available from the corresponding author on reasonable request.

## Abbreviations

CF: Cystic fibrosis
CLAD: Chronic lung allograft dysfunction
CT: Computed tomography
LTX: Lung transplant
NTM: Non-tuberculous mycobacteria
