# Supplementary material for: Outcome and prognostic factors after lung transplantation for bronchiectasis other than cystic fibrosis
Source: BMC Pulm Med. 2021 Aug 13;21:261. doi: 10.1186/s12890-021-01634-z (PMC8361737; doi:10.1186/s12890-021-01634-z)
Supplement: Supplementary file 1 — Additional file 1. Risk factors for mortality, development of CLAD and Pseudomonas colonization from multivariate Cox model. [file 12890_2021_1634_MOESM1_ESM.pdf]

Title

Outcome and prognostic factors after lung transplantation for bronchiectasis other than cystic fibrosis

Authors

Takashi Hirama¶[1,2], Fumiko Tomiyama[1], Hirotsugu Notsuda[1], Tatsuaki Watanabe[1], Yui Watanabe[1], Hisashi Oishi[1], Yoshinori Okada[1,2]

Affiliation

1. Department of Thoracic Surgery, Institute of Development, Aging and Cancer, Tohoku University, Sendai, Miyagi, Japan
2. Division of Organ Transplantation, Tohoku University Hospital, Sendai, Miyagi, Japan

¶corresponding author

Supplemental data. Hazard ratio for risk factors for mortality, development of CLAD and *Pseudomonas* colonization from multivariate Cox model

	A. Risk factors for death			B. Risk factors for CLAD			C. Risk factors for *Pseudomonas* colonization		
**Covariate**	p-value	HR	95% CI	p-value	HR	95% CI	p-value	HR	95% CI
Recipient age at LTX	0.016	1.04	1.01-1.08	0.008	1.05	1.01-1.09	0.904	1.00	0.97-1.04
Recipient sex (male vs female)	0.796	1.10	0.54-2.25	0.074	1.96	0.94-4.09	0.453	1.36	0.61-3.07
LTX indication (bronchiectasis vs others)	0.742	1.62	0.09-28.69	0.400	0.35	0.03-4.05	0.282	6.34	0.22-183.6
History of pre-transplant *Pseudomonas*	0.892	0.83	0.06-12.36	0.522	1.97	0.25-15.69	0.779	0.64	0.03-14.48
Chronic sinusitis	0.969	1.02	0.32-3.30	0.075	2.77	0.90-8.51	0.948	1.05	0.27-4.04

	D. Risk factors for NTM colonization			E. Risk factors for *Aspergillus* colonization		
**Covariate**	p-value	HR	95% CI	p-value	HR	95% CI
Recipient age at LTX	0.283	1.03	0.98-1.08	0.544	1.01	0.97-1.06
Recipient sex (male vs female)	0.111	2.39	0.82-6.95	0.797	0.88	0.33-2.34
LTX indication (bronchiectasis vs others)	0.034	20.70	1.26-341.2	0.056	0.08	0.01-1.07
History of pre-transplant *Pseudomonas*	0.040	0.06	0.01-0.88	0.013	15.43	1.78-133.7
Chronic sinusitis	0.556	1.61	0.33-7.89	0.204	2.29	0.64-8.24

LTX, lung transplant; CLAD, chronic lung allograft dysfunction; CI, confidence interval; HR, hazard ratio; NTM, non-tuberculous mycobacteria
